# The phase transition of rubidium hydrogen carbonate, RbHCO_3_

**DOI:** 10.1107/S2056989017008271

**Published:** 2017-06-07

**Authors:** Carla Larvor, Berthold Stöger

**Affiliations:** a Département Mesures Physique, Institut Universitaire Téchnologique de Bordeaux, 15 Rue de Naudet, 33175 Gradignan, France; b X-Ray Centre, TU Wien, Getreidemarkt 9, A-1060 Vienna, Austria

**Keywords:** crystal structure, order/disorder transition, hydrogen carbonate

## Abstract

Rubidium hydrogen carbonate, RbHCO_3_, features an order/disorder phase transition at *T*_C_ = 245 K from the high-temperature (HT) disordered *C*2/*m* modification to the low-temperature (LT) *C*-1 modification. The crystal structures are characterized by [HCO_3_]_2_^2−^ pairs of hydrogen carbonate groups connected by strong hydrogen bonding. The [HCO_3_]_2_^2−^ pairs are connected by Rb^+^ cations into a three-dimensional network. In HT-RbHCO_3_, the hydrogen atom is disordered. In LT-RbHCO_3_, ordering of the hydrogen atom leads to a *translation­engleiche* symmetry reduction of index 2. The lost reflections and rotations are retained as twin operations.

## Abstract 2

Rubidium hydrogen carbonate shows an order/disorder phase transition with the high-temperature phase (HT) having monclinic and the low-temperature phase triclinic symmetry. A comparison is made with the related HT/LT structures of potassium hydrogen carbonate.

## Chemical context   

The crystal chemistry of partially protonated oxoanions of main group elements [*e.g.* hydrogen carbonates, (di)hydrogen phosphates, hydrogen sulfates *etc*] is characterized by the formation of strong hydrogen bonds. Topologically, the hydrogen-bonding network may lead to isolated units (*e.g.* pairs in KHCO_3_; Thomas *et al.*, 1974 ▸), infinite chains (*e.g.* NaHCO_3_; Sass & Scheuerman, 1962 ▸) or two-dimensional networks (*e.g.* CsH_2_PO_4_; Uesu & Kobayashi, 1976 ▸). Compounds with such extended hydrogen-bonded network structures may be useful as proton conductors (Kim *et al.*, 2015 ▸).

In many cases, at higher temperatures, the hydrogen atoms are dynamically disordered between the connected oxoanions. On cooling, the disorder is ‘frozen’, resulting in a reduction of symmetry (order/disorder phase transition). Such phase transitions are of technological importance, for example in the KH_2_PO_4_ (KDP) family of compounds and therefore have been studied extensively. At high temperatures, these compounds exist in a paraelectric tetra­gonal phase. On cooling below *T*_C_, they order into ortho­rhom­bic ferroelectrics. This kind of phase transition is likewise of theoretical inter­est, because it allows the study of proton quantum dynamics (Fillaux *et al.*, 2008 ▸).

From a crystallographic point of view, these phase trans­itions offer the potential to study group/subgroup relationships (Müller, 2013 ▸). Moreover, in the case of a reduction of point symmetry, the lost symmetry is typically retained as a twin operation, leading to inter­esting as well as challenging problems.

A well known example of a hydrogen-bonding order/disorder transition is potassium hydrogen carbonate, KHCO_3_ (Kashida & Yamamoto, 1990 ▸). Above *T*_C_ = 318 K, it crystallizes in a monoclinic *C*2/*m* phase featuring disorder of the hydrogen atom (Fillaux *et al.*, 2008 ▸). On cooling, it transforms into an ordered *P*2_1_/*a* phase (Thomas *et al.*, 1974 ▸). Rubidium hydrogen carbonate RbHCO_3_ shows an analogous phase transition at *T*_C_ = 245 K, which has been thoroughly studied by NMR spectroscopy (Odin, 2004 ▸). The published structural data, on the other hand, leave much to be desired. A structure model of the high-temperature (HT) modification in the *C*2 space group has been provided by Kim (1969 ▸). The structure was later redetermined by Cirpus (1997 ▸), establishing the correct space group *C*2/*m* and isotypism with KHCO_3_. The lattice metrics of the low-temperature (LT) modification were identified as triclinic by Müller & Roth (2005 ▸). Although a model was refined by these authors, structural data were not deposited. To fill this gap, in this communication we report detailed structural data of the LT modification of RbHCO_3_ which were derived from a twinned crystal. We also redetermined the structure of the HT modification. The phase transition is discussed in detail and contrasted to the structural changes observed in KHCO_3_.
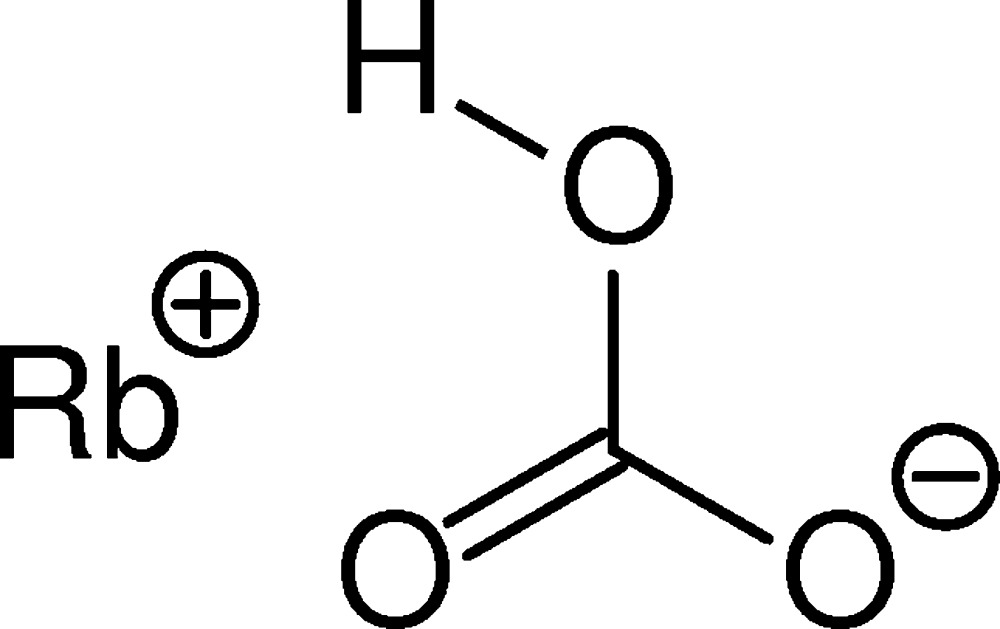


## Structural commentary   

### General   

**Figure 1:**
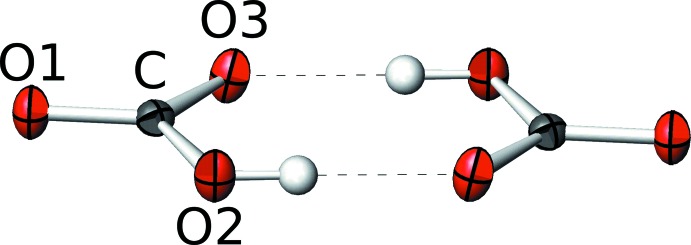
[HCO_3_]_2_^2−^ pair in LT-RbHCO_3_ connected by strong hydrogen bonding. C and O atoms are represented by grey and red ellipsoids drawn at the 75% probability levels and H atoms by white spheres of arbitrary radius. Hydrogen bonding is indicated by dashed lines.

The structure model of HT-RbHCO_3_ (*C*2/*m*) is in good agreement with that of Cirpus (1997 ▸). The crystal structures of HT-RbHCO_3_ (*C*2/*m*) and LT-RbHCO3 (C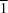) are closely related. The central building blocks are pairs of HCO_3_^−^ anion groups, which are connected by strong hydrogen bonds (Fig. 1 ▸, Tables 1 ▸ and 2 ▸). The prime cause for the order/disorder phase transition is the dynamic behaviour of the protons in these pairs. In HT-RbHCO_3_, they are dynamically disordered, resulting in a short [C—O1: 1.237 (4) Å] and two symmetry-equivalent inter­mediate [2×C—O2: 1.307 (3) Å] C—O bonds. The [HCO_3_]_2_^2−^ pair accordingly possesses 2/*m* point group symmetry. On cooling, the protons cannot overcome the tunneling barrier and are attached to distinct O atoms. In consequence, the point group symmetry of the [HCO_3_]_2_^2−^ pair is reduced to 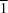. There are two short [C—O1: 1.241 (4) Å, C—O3: 1.270 (4) Å] and one longer [C—O2: 1.349 (4) Å] bond, as is characteristic for partially hydrogenated oxoanions. In both cases, the HCO_3_^−^ group is flat [distance of the C atom to the plane defined by the three O atoms: 0.000 (4) Å (HT) and 0.007 (3) Å (LT)], in accordance with literature data (Zemann, 1981 ▸).

**Figure 2:**
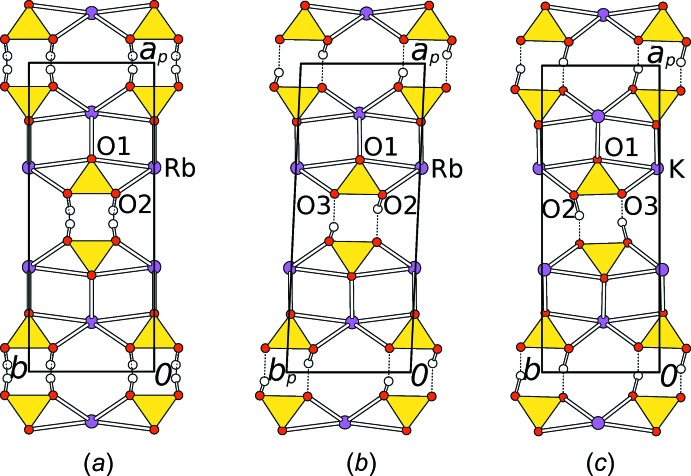
The crystal structures of (*a*) HT-RbHCO_3_, (*b*) LT-RbHCO_3_ and (*c*) LT-KHCO_3_ viewed down [001]. Rb/K atoms are represented by purple spheres, O atoms by red spheres, H atoms by colourless spheres and CO_3_^2−^ groups by yellow triangles.

**Figure 4:**
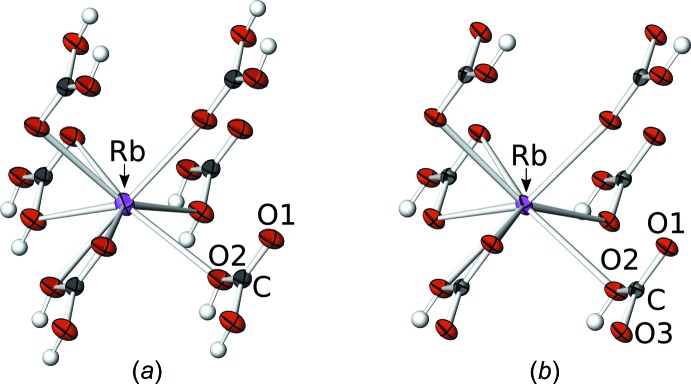
Coordination environment of the Rb^+^ cations in (*a*) HT-RbHCO_3_ and (*b*) LT-RbHCO_3_. Atoms as in Fig. 1 ▸, Rb^+^ cation are purple.

The [HCO_3_]_2_^2−^ pairs are connected by the Rb^+^ cations into a three-dimensional network (Figs. 2 ▸ and 3 ▸). The Rb^+^ cations are located on the reflection plane of the *C*2/*m* group (HT-RbHCO_3_) or on general positions (LT-RbHCO_3_). They are connected to six carbonate groups, two of which coordinate in a bidentate manner, the four others *via* one O atom (Fig. 4 ▸). Thus, in total, the Rb^+^ cations are coordinated by eight O atoms with bond lengths in the ranges 2.869 (3)–3.0662 (12) Å (HT-RbHCO_3_) and 2.865 (3)–3.101 (2) Å (LT-RbHCO_3_).

### Symmetry reduction and relationship to KHCO_3_   

Whereas the HT-KHCO_3_ and HT-RbHCO_3_ phases are isotypic, the corresponding LT phases are not. To understand the different behaviour on cooling, it is useful to consider the structures as being made up of layers of [HCO_3_]_2_^2−^ pairs parallel to (100). In the HT phases, these layers possess the *p*12/*m*1 layer group symmetry. On cooling, owing to the ordering of the protons, the *m*_[010]_ operation is lost. All [HCO_3_]_2_^2−^ pairs are rotated in the same direction about [001], resulting in layers with *p* 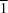 symmetry.

Thus, the lower symmetry layers may appear in one out of two orientations with respect to the [010] direction. In the LT-RbHCO_3_ phase, all layers feature the same orientation. Adjacent layers are related by translation symmetry (as in the *C*2/*m* HT phase) and therefore the translation lattice is retained. The symmetry loss concerns only the point symmetry. Since one out of two symmetry operations is retained (*viz*. the translations and inversions), the symmetry reduction is of the *t*2 kind, where *t* stands for *translationengleiche* and 2 for the index of 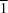 in 2/*m* (Müller, 2013 ▸).

In the LT-KHCO_3_ phase, on the other hand, layers feature alternating orientation with respect to [010]. The layers are split in two sets of translationally equivalent layers. The translation lattice is therefore reduced by an index of two, which here corresponds to a change of the Bravais lattice (*mC* to *mP*) while retaining the volume of the cell in the (more convenient but non-standard) centred setting. In return, adjacent layers are related by an *a* glide reflection. Thus, the point symmetry 2/*m* is retained. The symmetry reduction is therefore of the *k*2 kind, where *k* stands for *klassengleiche* (Müller, 2013 ▸).

**Figure 5:**
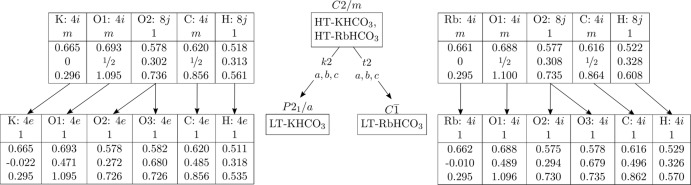
Bärnighausen family tree (Bärnighausen, 1980 ▸) representing the symmetry reduction from the HT-KHCO_3_ and RbHCO_3_ modifications to their LT modifications. Coordinates of the KHCO_3_ modifications were adapted from Fillaux *et al.* (2008 ▸) and Thomas *et al.* (1974 ▸).

The structural relationships of the HT and LT phases of RbHCO_3_ and KHCO_3_ are represented in a Bärnighausen family tree in Fig. 5 ▸. The atomic labelling and coordinates of the KHCO_3_ modifications were adapted from the original literature (Fillaux *et al.*, 2008 ▸; Thomas *et al.*, 1974 ▸) to be comparable to the data presented here. Note that the fractional coordinates of all four phases depicted in Fig. 5 ▸ are remarkably similar.

The atoms on the reflection planes in the HT phases are located on general positions in the LT phases. The O2 atom, which is located on a general position in the HT phase, is split into two positions in the LT phase. In contrast, the position of the H atom, which is also located on a general position in the HT modification, is not split. Instead, its occupancy is raised from 0.5 to 1.

### Twinning   

Phase transitions are one of the classical causes of twinning (Hahn & Klapper, 2006 ▸; Stöger *et al.*, 2016 ▸). If the point symmetry of the structure is reduced, the lost operations may be retained as twin operations. Indeed, the crystals of RbHCO_3_ were all systematically twinned below *T*_C_. The crystal under investigation was made up of two domains related by *m*_[010]_ and equivalently 2_[010]_, which corresponds precisely to the second coset in the coset decomposition of 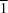 in 2/*m*. The twin volume ratio was determined by the *TWINABS* software as 51.3:48.7, which compares well to the volume ratio obtained from the (abandoned, see Section 3.3) refinement against HKLF 5 style data [52.0:48.0 (4)].

**Figure 6:**
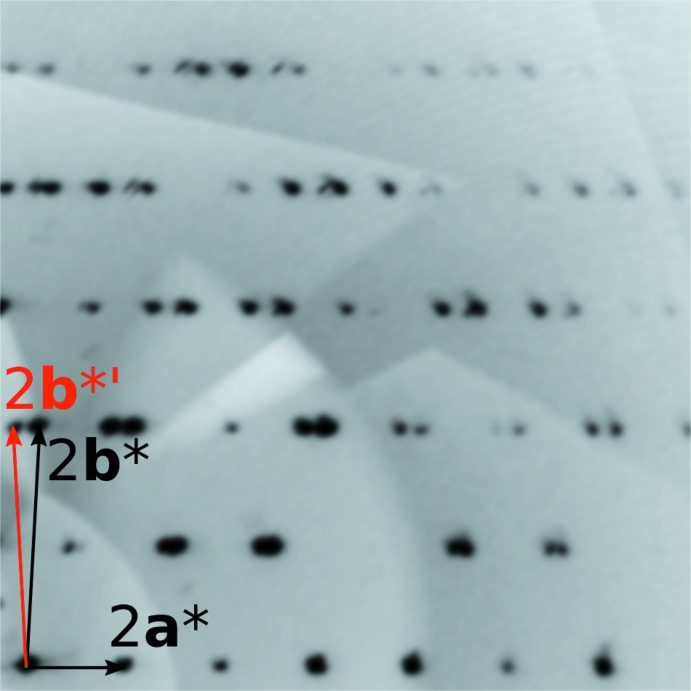
Part of the *hk*2 plane in reciprocal space of LT-RbHCO_3_ reconstructed from CCD data. The reciprocal basis vectors of the two twin domains are indicated.

Since the transformation into the triclinic *C* 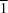 LT phase results in a substantial increase of the *γ* angle to *γ* = 92.748 (9)°, the diffraction spots at higher *k* indices are clearly separated (Fig. 6 ▸). Such a twin cannot be treated as a twin by pseudo-merohedry. The *α* angle, on the other hand, deviates only slightly from the monoclinic metrics [*α* = 89.343 (4)°]. The lattice of the layers therefore is pseudo-rectangular, which is consistent with the crystallo-chemical considerations above.

In KHCO_3_, the HT and LT phase feature the same point symmetry 2/*m*. Stacking faults therefore do not result in twinning but in anti­phase domains (Wondratschek & Jeitschko, 1976 ▸). These kinds of domains are significantly more difficult to qu­antify using X-ray diffraction.

## Experimental   

### Synthesis and crystallization   

Large crystals of RbHCO_3_ were grown by dissolving commercial ‘Rb_2_CO_3_’ (actually the sesquihydrate according to powder X-ray diffraction) in a small qu­antity of water followed by evaporation of the solution overnight at *ca* 295 K.

### Data collection   

Crystals were cut to sizes suitable for single crystal diffraction with a razor blade. Abrupt cooling of the crystals to below the phase-transition temperature by immersion into a cooled N_2_ stream led to fourfold splitting of reflections as described by Müller & Roth (2005 ▸). Data reduction was successful using four orientation matrices and a reasonable structure model could be obtained. Nevertheless, the quality of the refinement was deemed not optimal (notably, the hydrogen atoms could not be located). From structural reasoning, only two domains are expected (see Section 2.2). The higher number of domains was therefore attributed to a cracking of the crystal under thermal stress. Therefore, a data collection was first performed above *T*_C_ at 270 K. Then, the crystal was slowly (2 K h^−1^) cooled to 200 K and a full sphere of reciprocal space was collected with fine slicing. The first scan was discarded because it contained distinct reflections from the HT phase as well as two LT domains. The data set obtained from the remaining scans featured only the two expected LT twin domains.

### Data processing   

Data of the HT modification was subjected to routine processing using *SAINT* and *SADABS* (Bruker, 2016 ▸). For the LT phase, reflections of both domains were separated and reduced to intensity data using overlap information. An absorption correction was applied using the *TWINABS* (Bruker, 2016 ▸) software. This software outputs ‘detwinned’ conventional data (HKLF 4 style), usually used for structure solution and data with overlap information (HKLF 5 style). Surprisingly, the detwinned data set resulted in significantly better refinements. Not only were the residuals lower by two percentage points, additionally only in the detwinned data could the hydrogen atoms be located and refined. Therefore the discussion is based on the refinement using the detwinned data set.

### Structure solution and refinement   

An initial model of the HT modification was adapted from the data of Cirpus (1997 ▸). The structure of the LT modification was solved using the dual-space approach implemented in *SHELXT* (Sheldrick, 2015 ▸). Atomic coordinates and labelling were adapted to be analogous to those of the HT modification. The non-standard *C* 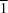 setting of the *P* 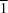 space group was chosen to facilitate comparison with the HT modification [lattice basis transformation from *P* 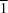 to *C* 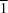: (**a**_*C*_, **b**_*C*_, **c** ***_C_***) = (**b**_*P*_ + 2**c**_*P*_, −**b**_*P*_, **a**_*P*_)]. The structure models were refined against *F* ^2^ with *JANA2006* (Petříček *et al.*, 2014 ▸). The hydrogen atoms were located in difference Fourier maps and the O—H distances restrained to 0.850 (1) Å. Crystal data, data collection and structure refinement details are summarized in Table 3 ▸. 

## Supplementary Material

Crystal structure: contains datablock(s) HT_RbHCO3, LT_RbHCO3, global. DOI: 10.1107/S2056989017008271/pk2601sup1.cif

Structure factors: contains datablock(s) HT_RbHCO3. DOI: 10.1107/S2056989017008271/pk2601HT_RbHCO3sup2.hkl

Structure factors: contains datablock(s) LT_RbHCO3. DOI: 10.1107/S2056989017008271/pk2601LT_RbHCO3sup3.hkl

CCDC references: 1554022, 1554021

Additional supporting information:  crystallographic information; 3D view; checkCIF report

## Figures and Tables

**Figure 3:**
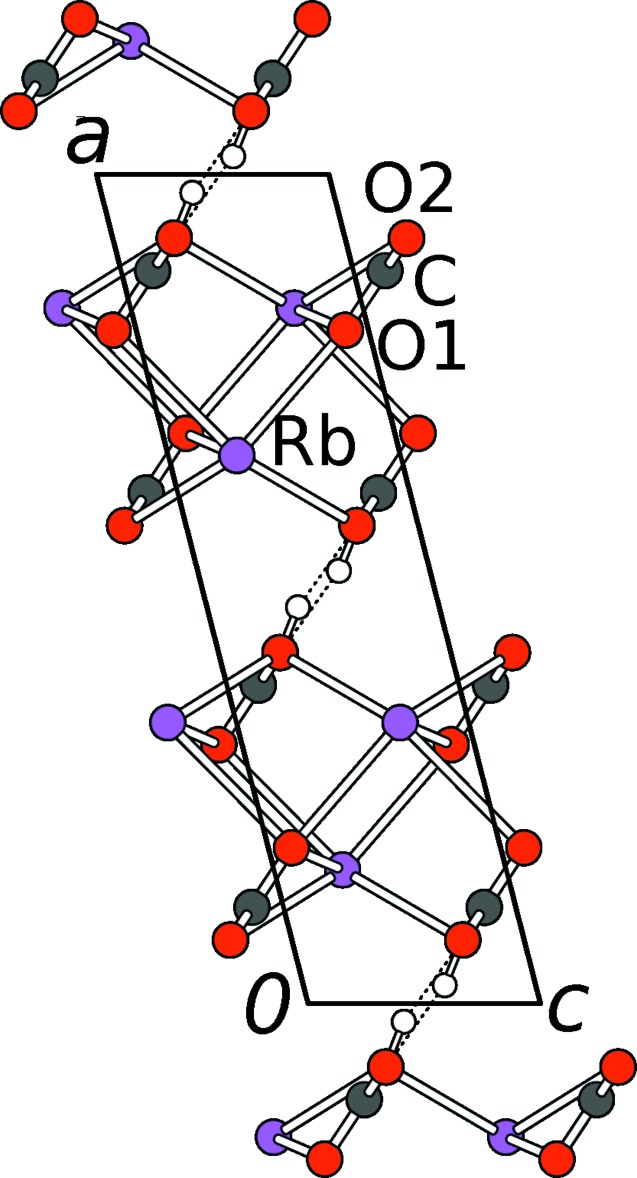
The crystal structure of HT-RbHCO_3_ viewed down [010]. Atoms as in Fig. 2 ▸, C atoms are grey.

**Table 1:** Hydrogen-bond geometry (Å, °) for HT-RbHCO3

*D*—H⋯*A*	*D*—H	H⋯*A*	*D*⋯*A*	*D*—H⋯*A*
O2—H⋯O2^i^	0.85 (4)	1.75 (4)	2.571 (3)	162 (6)

Symmetry code: (i) 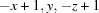.

**Table 2:** Hydrogen-bond geometry (Å, °) for LT-RbHCO3

*D*—H⋯*A*	*D*—H	H⋯*A*	*D*⋯*A*	*D*—H⋯*A*
O2—H⋯O3^i^	0.85 (3)	1.75 (3)	2.582 (3)	165 (4)

Symmetry code: (i) 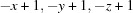.

**Table 3:** Experimental details

	HT-RbHCO_3_	LT-RbHCO_3_
Crystal data		
Chemical formula	RbHCO_3_	RbHCO_3_
*M*_r_	146.5	146.5
Crystal system, space group	Monoclinic, *C*2/*m*	Triclinic, *C* 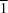
Temperature (K)	270	200
*a*, *b*, *c* (Å)	14.807 (3), 5.8216 (12), 4.0217 (9)	14.945 (3), 5.8212 (9), 3.9699 (6)
α, β, γ (°)	90, 104.321 (5), 90	89.343 (4), 104.096 (4), 92.748 (9)
*V* (Å^3^)	335.91 (12)	334.59 (9)
*Z*	4	4
Radiation type	Mo *K*α	Mo *K*α
μ (mm^−1^)	14.54	14.60
Crystal size (mm)	0.43 × 0.18 × 0.09	0.43 × 0.18 × 0.09

Data collection		
Diffractometer	Bruker Kappa APEXII CCD	Bruker Kappa APEXII CCD
Absorption correction	Multi-scan (*SADABS*; Bruker, 2016 ▸)	Multi-scan (*TWINABS*; Bruker, 2016 ▸)
*T*_min_, *T*_max_	0.05, 0.27	0.05, 0.27
No. of measured, independent and observed [*I* > 3σ(*I*)] reflections	3067, 672, 544	8005, 1215, 1029
*R*_int_	0.051	0.059
(sin θ/λ)_max_ (Å^−1^)	0.766	0.760

Refinement		
*R*[*F* ^2^ > 2σ(*F* ^2^)], *wR*(*F* ^2^), *S*	0.029, 0.066, 1.26	0.038, 0.075, 1.50
No. of reflections	672	1215
No. of parameters	32	50
No. of restraints	1	1
H-atom treatment	H atoms treated by a mixture of independent and constrained refinement	H atoms treated by a mixture of independent and constrained refinement
Δρ_max_, Δρ_min_ (e Å^−3^)	0.86, −0.96	1.88, −1.81

Computer programs: *APEX3* and *SAINT-Plus* (Bruker, 2016 ▸), *SHELXT* (Sheldrick, 2015 ▸), *JANA2006* (Petříček *et al.*, 2014 ▸), *ATOMS* (Dowty, 2006 ▸) and *publCIF* (Westrip, 2010 ▸).

## supplementary crystallographic information

### (HT_RbHCO3) Crystal data

**Table:**

RbHCO_3_	*F*(000) = 272
*M**_r_* = 146.5	*D*_x_ = 2.897 Mg m^−^^3^
Monoclinic, *C*2/*m*	Mo *K*α radiation, λ = 0.71073 Å
Hall symbol: -C 2y	Cell parameters from 1945 reflections
*a* = 14.807 (3) Å	θ = 2.8–32.6°
*b* = 5.8216 (12) Å	µ = 14.54 mm^−^^1^
*c* = 4.0217 (9) Å	*T* = 270 K
β = 104.321 (5)°	Rod, colourless
*V* = 335.91 (12) Å^3^	0.43 × 0.18 × 0.09 mm
*Z* = 4	

### (HT_RbHCO3) Data collection

**Table:**

Bruker KAPPA APEXII CCD diffractometer	544 reflections with *I* > 3σ(*I*)
Radiation source: X-ray tube	*R*_int_ = 0.051
ω– and φ–scans	θ_max_ = 33.0°, θ_min_ = 2.8°
Absorption correction: multi-scan (SADABS; Bruker, 2016)	*h* = −22→20
*T*_min_ = 0.05, *T*_max_ = 0.27	*k* = −8→8
3067 measured reflections	*l* = −6→6
672 independent reflections	

### (HT_RbHCO3) Refinement

**Table:**

Refinement on *F*^2^	0 constraints
*R*[*F*^2^ > 2σ(*F*^2^)] = 0.029	H atoms treated by a mixture of independent and constrained refinement
*wR*(*F*^2^) = 0.066	Weighting scheme based on measured s.u.'s *w* = 1/(σ^2^(*I*) + 0.0004*I*^2^)
*S* = 1.26	(Δ/σ)_max_ = 0.011
672 reflections	Δρ_max_ = 0.86 e Å^−^^3^
32 parameters	Δρ_min_ = −0.96 e Å^−^^3^
1 restraint	

### (HT_RbHCO3) Fractional atomic coordinates and isotropic or equivalent isotropic displacement parameters (Å^2^)

**Table:**

	*x*	*y*	*z*	*U*_iso_*/*U*_eq_	Occ. (<1)
Rb	0.66119 (3)	0	0.29537 (9)	0.02714 (13)	
O1	0.6878 (2)	0.5	1.0996 (8)	0.0324 (10)	
O2	0.57663 (15)	0.3079 (3)	0.7353 (5)	0.0304 (7)	
C	0.6159 (3)	0.5	0.8641 (9)	0.0210 (10)	
H	0.522 (2)	0.328 (12)	0.608 (16)	0.04 (2)*	0.5

### (HT_RbHCO3) Atomic displacement parameters (Å^2^)

**Table:**

	*U*^11^	*U*^22^	*U*^33^	*U*^12^	*U*^13^	*U*^23^
Rb	0.0363 (3)	0.02090 (17)	0.0221 (2)	0	0.00320 (15)	0
O1	0.0244 (16)	0.0332 (14)	0.0343 (16)	0	−0.0030 (13)	0
O2	0.0332 (13)	0.0173 (8)	0.0342 (11)	0.0018 (7)	−0.0040 (9)	−0.0016 (7)
C	0.0231 (19)	0.0186 (14)	0.0217 (17)	0	0.0062 (14)	0

### (HT_RbHCO3) Geometric parameters (Å, º)

**Table:**

Rb—O1^i^	2.869 (3)	Rb—O2^vi^	3.001 (2)
Rb—O1^ii^	3.046 (3)	Rb—C^iii^	3.370 (2)
Rb—O1^iii^	3.0662 (12)	Rb—C^iv^	3.370 (2)
Rb—O1^iv^	3.0662 (12)	O1—C	1.237 (4)
Rb—O2^iv^	2.908 (2)	O2—C	1.307 (3)
Rb—O2^v^	2.908 (2)	O2—H	0.85 (4)
Rb—O2	3.001 (2)		
			
O1^i^—Rb—O2^iv^	138.83 (5)	Rb^vii^—O1—C	172.6 (3)
O1^i^—Rb—O2	81.45 (7)	Rb—O2—Rb^viii^	85.75 (5)
O1^i^—Rb—O2^v^	138.83 (5)	Rb—O2—C	122.4 (2)
O1^i^—Rb—O2^vi^	81.45 (7)	Rb—O2—H	102 (4)
O2^iv^—Rb—O2	85.75 (6)	Rb^viii^—O2—C	98.99 (16)
O2^iv^—Rb—O2^v^	76.10 (5)	Rb^viii^—O2—H	135 (4)
O2^iv^—Rb—O2^vi^	131.47 (6)	C—O2—H	113 (5)
O2—Rb—O2^v^	131.47 (6)	O1—C—O2	121.17 (15)
O2—Rb—O2^vi^	73.35 (6)	O1—C—O2^ix^	121.17 (15)
O2^v^—Rb—O2^vi^	85.75 (6)	O2—C—O2^ix^	117.7 (3)

Symmetry codes: (i) −*x*+3/2, *y*−1/2, −*z*+2; (ii) −*x*+3/2, *y*−1/2, −*z*+1; (iii) *x*, *y*−1, *z*−1; (iv) *x*, *y*, *z*−1; (v) *x*, −*y*, *z*−1; (vi) *x*, −*y*, *z*; (vii) −*x*+3/2, *y*+1/2, −*z*+2; (viii) *x*, *y*, *z*+1; (ix) *x*, −*y*+1, *z*.

### (HT_RbHCO3) Hydrogen-bond geometry (Å, º)

**Table:**

*D*—H···*A*	*D*—H	H···*A*	*D*···*A*	*D*—H···*A*
O2—H···O2^x^	0.85 (4)	1.75 (4)	2.571 (3)	162 (6)

Symmetry code: (x) −*x*+1, *y*, −*z*+1.

### (LT_RbHCO3) Crystal data

**Table:**

RbHCO_3_	*Z* = 4
*M**_r_* = 146.5	*F*(000) = 272
Triclinic, *C*1	*D*_x_ = 2.908 Mg m^−^^3^
Hall symbol: -C 1	Mo *K*α radiation, λ = 0.71073 Å
*a* = 14.945 (3) Å	Cell parameters from 1945 reflections
*b* = 5.8212 (9) Å	θ = 2.8–32.6°
*c* = 3.9699 (6) Å	µ = 14.60 mm^−^^1^
α = 89.343 (4)°	*T* = 200 K
β = 104.096 (4)°	Rod, colourless
γ = 92.748 (9)°	0.43 × 0.18 × 0.09 mm
*V* = 334.59 (9) Å^3^	

### (LT_RbHCO3) Data collection

**Table:**

Bruker KAPPA APEXII CCD diffractometer	1029 reflections with *I* > 3σ(*I*)
Radiation source: X-ray tube	*R*_int_ = 0.059
ω– and φ–scans	θ_max_ = 32.7°, θ_min_ = 2.8°
Absorption correction: multi-scan (TWINABS; Bruker, 2016)	*h* = −6→22
*T*_min_ = 0.05, *T*_max_ = 0.27	*k* = −8→8
8005 measured reflections	*l* = −6→5
1215 independent reflections	

### (LT_RbHCO3) Refinement

**Table:**

Refinement on *F*^2^	0 constraints
*R*[*F*^2^ > 2σ(*F*^2^)] = 0.038	H atoms treated by a mixture of independent and constrained refinement
*wR*(*F*^2^) = 0.075	Weighting scheme based on measured s.u.'s *w* = 1/(σ^2^(*I*) + 0.0004*I*^2^)
*S* = 1.50	(Δ/σ)_max_ = 0.018
1215 reflections	Δρ_max_ = 1.88 e Å^−^^3^
50 parameters	Δρ_min_ = −1.81 e Å^−^^3^
1 restraint	

### (LT_RbHCO3) Fractional atomic coordinates and isotropic or equivalent isotropic displacement parameters (Å^2^)

**Table:**

	*x*	*y*	*z*	*U*_iso_*/*U*_eq_	
Rb	0.66221 (2)	−0.00995 (4)	0.29542 (8)	0.01812 (10)	
O1	0.68811 (18)	0.4885 (4)	1.0957 (7)	0.0232 (8)	
O2	0.57544 (19)	0.2938 (4)	0.7298 (7)	0.0218 (8)	
O3	0.57832 (19)	0.6790 (4)	0.7345 (6)	0.0219 (8)	
C	0.6160 (2)	0.4961 (5)	0.8620 (8)	0.0153 (9)	
H	0.529 (2)	0.326 (7)	0.570 (10)	0.037 (13)*	

### (LT_RbHCO3) Atomic displacement parameters (Å^2^)

**Table:**

	*U*^11^	*U*^22^	*U*^33^	*U*^12^	*U*^13^	*U*^23^
Rb	0.02362 (19)	0.01426 (16)	0.01495 (15)	0.00035 (9)	0.00184 (11)	0.00014 (9)
O1	0.0190 (14)	0.0226 (11)	0.0236 (13)	0.0006 (8)	−0.0032 (10)	0.0001 (8)
O2	0.0227 (14)	0.0118 (10)	0.0266 (13)	0.0014 (8)	−0.0022 (11)	−0.0019 (8)
O3	0.0256 (14)	0.0128 (10)	0.0223 (12)	0.0003 (8)	−0.0033 (10)	0.0007 (7)
C	0.0155 (16)	0.0151 (13)	0.0155 (14)	−0.0005 (10)	0.0047 (12)	−0.0003 (9)

### (LT_RbHCO3) Geometric parameters (Å, º)

**Table:**

Rb—O1^i^	3.031 (2)	Rb—O3^v^	2.944 (3)
Rb—O1^ii^	3.015 (3)	Rb—C^iv^	3.324 (3)
Rb—O1^iii^	2.865 (3)	Rb—C^i^	3.408 (3)
Rb—O1^iv^	3.101 (2)	O1—C	1.241 (4)
Rb—O2^i^	2.926 (2)	O2—C	1.349 (4)
Rb—O2	3.027 (3)	O2—H	0.85 (3)
Rb—O3^iv^	2.885 (2)	O3—C	1.270 (4)
			
O1^i^—Rb—O1^ii^	73.01 (7)	O3^iv^—Rb—O3^v^	85.85 (7)
O1^i^—Rb—O1^iii^	94.75 (6)	Rb^vi^—O1—Rb^ii^	106.99 (8)
O1^i^—Rb—O2^i^	43.56 (6)	Rb^vi^—O1—Rb^iii^	85.25 (6)
O1^i^—Rb—O2	70.67 (7)	Rb^vi^—O1—C	96.77 (18)
O1^i^—Rb—O3^iv^	116.17 (7)	Rb^ii^—O1—Rb^iii^	84.89 (7)
O1^i^—Rb—O3^v^	144.31 (8)	Rb^ii^—O1—C	103.4 (2)
O1^ii^—Rb—O1^iii^	84.89 (8)	Rb^iii^—O1—C	170.4 (3)
O1^ii^—Rb—O2^i^	81.38 (7)	Rb—O2—Rb^vi^	83.64 (6)
O1^ii^—Rb—O2	140.08 (6)	Rb—O2—C	122.1 (2)
O1^ii^—Rb—O3^iv^	80.76 (7)	Rb—O2—H	97 (3)
O1^ii^—Rb—O3^v^	141.28 (7)	Rb^vi^—O2—C	98.99 (17)
O1^iii^—Rb—O2^i^	138.29 (6)	Rb^vi^—O2—H	149 (3)
O1^iii^—Rb—O2	82.17 (7)	C—O2—H	107 (3)
O1^iii^—Rb—O3^iv^	139.80 (7)	Rb^vii^—O3—Rb^viii^	85.85 (6)
O1^iii^—Rb—O3^v^	82.33 (7)	Rb^vii^—O3—C	121.9 (2)
O2^i^—Rb—O2	83.64 (7)	Rb^viii^—O3—C	98.73 (18)
O2^i^—Rb—O3^iv^	76.08 (6)	O1—C—O2	117.2 (3)
O2^i^—Rb—O3^v^	130.12 (8)	O1—C—O3	125.2 (3)
O2—Rb—O3^iv^	130.57 (7)	O2—C—O3	117.6 (3)
O2—Rb—O3^v^	73.70 (7)		

Symmetry codes: (i) *x*, *y*, *z*−1; (ii) −*x*+3/2, −*y*+1/2, −*z*+1; (iii) −*x*+3/2, −*y*+1/2, −*z*+2; (iv) *x*, *y*−1, *z*−1; (v) *x*, *y*−1, *z*; (vi) *x*, *y*, *z*+1; (vii) *x*, *y*+1, *z*; (viii) *x*, *y*+1, *z*+1.

### (LT_RbHCO3) Hydrogen-bond geometry (Å, º)

**Table:**

*D*—H···*A*	*D*—H	H···*A*	*D*···*A*	*D*—H···*A*
O2—H···O3^ix^	0.85 (3)	1.75 (3)	2.582 (3)	165 (4)

Symmetry code: (ix) −*x*+1, −*y*+1, −*z*+1.
